# A Mechanics Based Surface Image Interpretation Method for Multifunctional Nanocomposites

**DOI:** 10.3390/nano9111578

**Published:** 2019-11-07

**Authors:** Brina J. Blinzler, Ragnar Larsson, Karolina Gaska, Roland Kádár

**Affiliations:** 1 Division of Material and Computational Mechanics, Department of Industrial and Materials Science, Chalmers University of Technology, SE-412 96 Göteborg, Sweden; ragnar.larsson@chalmers.se; 2 Division of Engineering Materials, Department of Industrial and Materials Science, Chalmers University of Technology, SE-412 96 Göteborg, Sweden or karolina.gaska@bristol.ac.uk (K.G.); roland.kadar@chalmers.se (R.K.)

**Keywords:** graphene, multifunctional composites, nanocomposite, computational modeling

## Abstract

Graphene nanosheets and thicker graphite nanoplatelets are being used as reinforcement in polymeric materials to improve the material properties or induce new functional properties. By improving dispersion, de-agglomerating the particles, and ensuring the desired orientation of the nano-structures in the matrix, the microstructure can be tailored to obtain specific material properties. A novel surface image assisted modeling framework is proposed to understand functional properties of the graphene enhanced polymer. The effective thermal and mechanical responses are assessed based on computational homogenization. For the mechanical response, the 2-D nanoplatelets are modeled as internal interfaces that store energy for membrane actions. The effective thermal response is obtained similarly, where 2-D nanoplatelets are represented using regions of high conductivity. Using the homogenization simulation, macroscopic stiffness properties and thermal conductivity properties are modeled and then compared to the experimental data. The proposed surface image assisted modeling yields reasonable effective mechanical and thermal properties, where the Kapitza effect plays an important part in effective thermal properties.

## 1. Introduction

Graphene possesses a remarkable range of superior properties including mechanical, thermal, electrical, gas barrier, high surface area, and others, which makes it an important candidate as a reinforcement for polymers [1,2]. The properties of graphene-based polymer nanocomposites are strongly dependent on graphene preparation, incorporation method into the matrix polymer, and finally morphological control during processing. Two important aspects in composite response are stiffness and thermal conductivity. Alongside the experimental evidence, a stochastically-based predictive model is required to complete the understanding of the increased stiffness due to the interaction between the constituents of the nanocomposite.

The mechanical response of graphene polymer nanocomposites has been studied extensively experimentally [3,4,5]. Current mechanical analysis methods use analytical methods with an empirical basis such as formulated by Young et al. [6]. These analysis methods formulate an effective in-plane modulus of the nano-structure reinforcement using the shear lag theory for shear stress transfer between the constituent surfaces [7]. The analytical methodology also relies on a micromechanics theory known as the rule of mixtures (ROM) to homogenize the constituent responses in the overall composite material. Difficulties arise with analytical homogenization due to the inherent planar nature of the graphene and graphite nanoplatelet reinforcement. There have also been a number of finite element-based analysis methods developed [8,9,10] which develop an artificial microstructure for 2D or 3D simulations.

Recent research has been directed at the development and analysis of graphene polymer composites with high thermal performance. A considerable effort has been made to develop fabrication techniques for these composites [11]. A number of analytical methods for predicting effective or homogenized thermal conductivity of nonspherical particulate composites are available [12,13]. These methods use average particle sizes and distributions to calculate the overall material response. The overall thermal response of graphene polymer nanocomposites has been studied by Gresil et al. [14]. In this study, the particles investigated are graphene nanosheet stacks that have between 10 and 20 layers (10 > N > 20) cf. [4]. We refer to this type of particle due to the thickness dimension as a Graphite nanoPlatelet (GnP). A three-dimensional computational method for a single GnP in a polymer bulk has been developed [15]. This single representative volume element (RVE) is computed and used to formulate a three-dimensional volume with a user defined distribution of particle size, shape, and orientation. For graphene-based nanocomposites, it has been shown that the thermal conductivity of the overall composite is highly dependent on the orientation, size, and shape of the nanoplatelets in the bulk material [14].

A number of digital image analysis techniques have been developed for use on two-dimensional images with particles or inclusions of interest. The open source image processing package Fiji [16] has a large variety of scientific image analysis tools for this purpose. One of the primary tools that allows for the detection of the inclusions of interest is the variety of auto threshold methods which dynamically binarize each image [17]. This dynamic binarization uses a feedback loop to optimize the threshold value before converting the image into the two parts: foreground and background. This method is well suited for analysis of large sets of images because it can compensate for slight irregularities.

In the present paper a finite element-based representation was developed to meet this need in modeling the multifunctional properties of these composites. This model is combined with an automated method for extracting 2-D nanoparticle dispersion, orientation, and length that can be used for large sets of samples. The stochastic approach utilizes experimental through-thickness scanning electron microscope (SEM) images of nanocomposites are used to create virtual representations of the experimental microstructure. These SEM images are systematically analyzed through a series of image analysis techniques used to reduce errors in nano-structure extraction, increase repeatability, and greatly reduce the time require to analyze large sets of samples. In the finite element model, the 2-D graphite nanoplatelets are modeled as internal interfaces that store energy for membrane strains of the GnP. From the homogenization analysis, macro level stiffness properties are simulated and compared to experimental results. Using a 2-D image and finite element representation of micrographs of low-density polyethylene (LDPE) embedded with (2-D) graphite nanoplatelets obtained via melt extrusion, the mechanical and thermal properties are assessed based on computational homogenization.

In this paper Section 2 the materials and methods, discusses the nanomaterials investigated and how they were prepared and tested. Section 3 the theory, discusses the image processing, the analysis method for the microstructure, the mechanical property calculation method, and the thermal property calculation method. Section 4 results, discusses the computational results of the mechanical and thermal calculations as well as their comparison with experimental results and existing methods. Finally, from the concluding remarks we arrive at the following implications of the paper: surface images can yield reasonable effective property estimates for these types of nanocomposites, both effective mechanical and thermal properties are dominated by the largest GnPs in the sample, and the Kapitza effect plays an important role in the prediction of effective thermal properties.

## 2. Materials and Methods

### 2.1. Materials

The material constituents of the composite include low density polyethylene (Borealis AB, Stenungsund, Sweden) and graphene nanoplatelets M5 purchased from XG Sciences (Lansing, MI, USA). More detailed information about used materials can be found in Gaska et al. [1].

### 2.2. Manufacturing

The performance and properties of polymer nanocomposites can depend to a significant degree on several of the previously outlined processing considerations. The interactions between the processing flow field, matrix, and filler are crucial for de-aggregating the particles, ensuring a good dispersion of the filler and inducing the desired orientation. Two-dimensional nanoparticles, e.g., Graphene/GnP, can be oriented in the flow direction starting with low shear rates (e.g., as low as 10^−2^ s^−1^ [1]). This can enable the creation of highly structured (thermoplastic in this case) polymer nanocomposites [1,18,19]. Such bulk nanocomposite morphologies exhibit anisotropic electrical and thermal properties with potential applications to field grading materials [18] and heat management components [1] as well as antibacterial properties [20]. A basic general performance requirement for nanocomposites is the filler’s mechanical reinforcing effect. In highly structured GnP-polymer nanocomposites, moderate improvements in Young’s modulus, the yield strength, and tensile strength were recorded with increasing filler concentration [1,19]. Higher values were recorded in the direction of the extrusion flow, compared to the perpendicular direction, likely due to the flow- induced molecular orientation [1]. A downside of improved mechanical properties is the loss of material ductility with increasing filler concentration [1,19].

A detailed description of the experimental procedures for obtaining highly structured graphene polymer nanocomposites can be found in Gaska et al. (2017) [1] and Gaska et al. (2017) [19,21].

### 2.3. Tensile Tests

The tensile tests were carried out according to the ISO 37-2. More detailed information can be found in Gaska et al. (2017) [1].

### 2.4. Scanning Electron Microscopy

Scanning Electron Microscopy (SEM) observations were carried out on cryo-fractured surfaces. All samples were etched using a potassium permanganate etchant typically used for polyolefin samples [1]. Thereafter, the samples were coated with approximately 5 nm thick gold layer. A LEO Ultra 55 high resolution SEM was used to investigate the morphology of the nanocomposites using an acceleration voltage of 5 kV.

### 2.5. Thermal Conductivity Tests

Thermal conductivity at room temperature was investigated by means of a transient heat source (Hot Disk Thermal Constant Analyzer TPS 2500 s), according to ISO 22007-2:2008. More detailed information can also be found in Gaska et al. [1].

## 3. Theory

### 3.1. Image Interpretation

The approach presented here utilizes experimental through-thickness scanning electron microscope (SEM) images of nanocomposites to create virtual representations of the experimental microstructure. The SEM images were analyzed systematically through a series of image analysis techniques used to reduce errors in 2-D nano-structure extraction, increase repeatability, and significantly reduce the time required to analyze large sets of samples. The open source image analysis tool Fiji is used which is an extension and compilation of ImageJ [16], Fourier, and other open source image analysis tools. The analysis steps were executed via a script suitable for batch processing of large numbers of files. There were five image analysis steps. First, the raw images were cropped to 30 × 30 μm randomly. Then Fiji’s built-in Auto Threshold was used with the Li Method [22], where Li’s minimum cross entropy thresholding method using the iterative algorithm was utilized. The Fiji’s Particle Analyzer was used to identify the nano-structures. Following this, the nano-structures shorter than 2 μm were removed. The reasoning for removal will be explained in more detail in the following section. To properly represent the microscopic fluctuation using finite elements, the Feret’s Diameter Method was used to approximate the remaining GnPs as line elements. The nano-structure location, length, and angle were then passed to the subsequent mesh creation scripts via text files.

**Figure 1:**
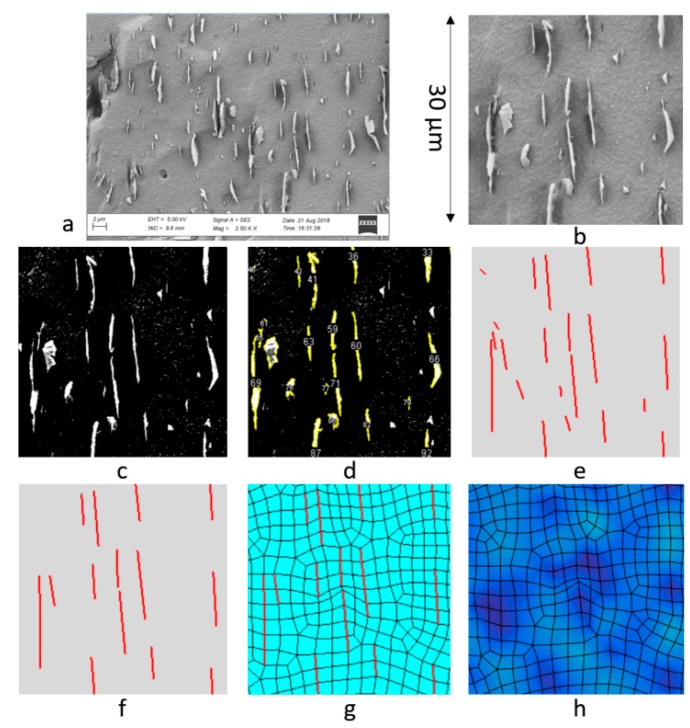
Homogenization of the elastic orthotropic E_11_ component in the drawing direction (here vertical direction) of the oriented low-density polyethylene (LDPE)/Graphite nanoPlatelet (GnP) nanocomposite: (**a**) experimental image [1], (**b**) trimmed image region of interest (30 µm × 30 µm), (**c**) threshold based binarization of image, (**d**) automatic detection of nano-structures, (**e**) linear approximation of nano-structures, (**f**) selected nano-structures of interest, (**g**) mesh with 1-D graphene representations, (**h**) deformation in the vertical (11) direction.

Results from the major steps of the image interpretation process can be seen in Figure 1a–f. Figure 1a displays an example of a raw image file. This file contains both the image and the scale of the image required to set the pixel to length scale for the subsequent calculations. Figure 1b shows a cropped surface image. The result of binarization via auto thresholding can be seen in Figure 1c. Li was the optimal threshold tool found for the set of image files out of the 17 auto threshold tools available in Fiji [16].

All files included in this paper were processed with the Li auto threshold [22]. The objects which meet the criteria defined in the particle analyzer are traced in yellow in Figure 1d. For the criteria used, the size was set to 0.10–4.00 and the circularity was set to 0.00–0.30. Figure 1e displays a virtual representation of the data extracted via the Feret’s diameter method. The larger nano-structures shown in Figure 1f were then used for the representation of the effective mechanical and thermal properties of nanocomposites. The systematic image interpretation method allows for quick processing of large numbers of images for stochastic variation of the polymer embedded GnPs. The resulting processed images were finite element discretized, which together with the mechanical/thermal modeling, were used for computational homogenization to obtain the effective properties. Both mechanical and thermal effective properties are homogenized, as discussed in Section 3.2 and Section 3.3 below.

### 3.2. Mechanical Property Calculations

#### 3.2.1. Effective In-Plane Stiffness of Graphene

It has been shown that there is a reduction in the resulting in-plane elastic modulus of graphene sheets when embedded in a polymer matrix as compared to neat graphene monolayers [3,4,5,6,21]. Among the main reasons is that the vast majority of commercial graphene for composites are far from monolayers and there could be substantial aggregation of the fillers. An effective in-plane elastic modulus [6] was computed to simulate overall effective response of the composite. This effective modulus is highly dependent on the elastic modulus of the matrix, the size of the dominate graphite nanosheets in the continuum, the number of monolayers, and the surface functionalization. It has been noted [2] that increases in mechanical stiffness response correlate with greater characteristic lengths of the reinforcement and with matrix compatible surface functionalization. In the computational homogenization, it was noted that the homogenized stiffness response was dominated by the largest graphite nanosheet in the 2D image. The effective in-plane stiffness was corelated using the experimental results of the elastic modulus in extrusion direction. The effective multiplier was used to calibrate the model, which is a ratio of the effective in-plane elastic modulus of the GnP to the elastic modulus of the matrix.

#### 3.2.2. Computational Homogenization of Mechanical Properties

In order to predict and complement the stiffness increase which has been experimentally observed of the reinforced matrix, a method that combines the image analysis presented above with computational homogenization was developed to assess the effective elastic response of the graphene enhanced polymer.

As alluded to in Figure 1f,g, the nano-structures of the images were embedded 1D elements, cf. in [20], whereas the neat PE resin represents the bulk of the image area. In order to predict the theoretical increase in the stiffness of the overall composite, a finite element model-based method was developed. The effective elastic representation of the 2-D nano-structure-enhanced composite was extracted from computational homogenization, cf. in [2,23] in this method. In this homogenization, the 2-D GnP embedded in the neat resin were considered as 1-D reinforcements resolved via the image analysis method presented above. 

**Figure 2:**
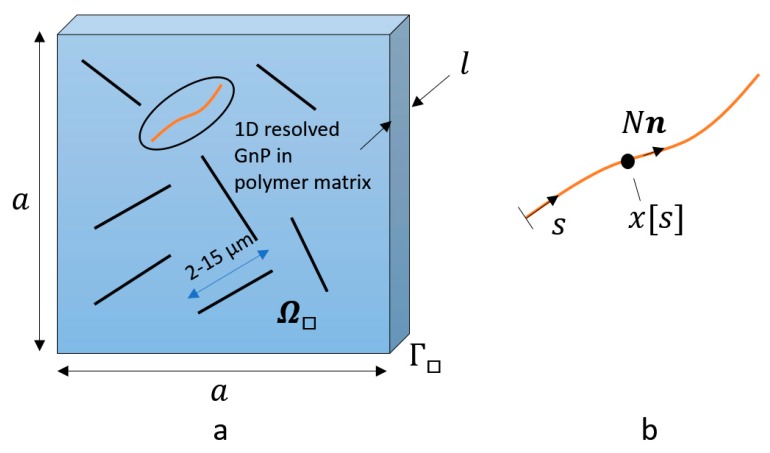
Sketch of 1-D nanostructures embedded in Representative Area Element (RAE) of neat resin. (**a**) principal 2D image of GnPs embedded in polymer matrix in the RAE region $\Omega_{□}$ with the boundary $\Gamma_{□}$. (**b**) Close-up of GnP considered as 1D line element with running natural coordinate *s* and tangent vector **n** of unit length.

Consider the 1-D resolved embedded GnP from the image in a Representative Area Element (RAE), as shown in Figure 2. The square region of the RAE is 𝛺□, of width and breadth 𝑎 = 30 μm, with embedded GnP considered as 1-D elements is shown in Figure 1b. The embedded 1-D elements have individual length (2–15 μm), thickness, orientation, and discrete locations given by the image analysis in Figure 1f. From the image analysis, the average GnP length and thickness are *l* and *t*, respectively. Considering the 1-D element in detail, which can be viewed in Figure 2b, the natural coordinate *s* is introduced, running along the 1-D structure with tangent vector 𝒏 in the integration interval 𝐼. The “stress” response is governed by the axial force 𝑁. It is assumed that the RAE plate has thickness *l* and that plane strain conditions holds.

For the homogenization, we consider the classical scheme of Suquet [24] stating the virtual work equivalence between the macroscopic and the microscopic fields in the RAE formulated as

(1) $$A\;\sigma_{m}:\;\delta \varepsilon_{m}=\int_{\Omega_{□}}^{}\sigma :\delta \varepsilon dS+\frac{1}{l}\;\sum_{i=1}^{n_{i}}{\left(\int_{I}^{}N\left[\varepsilon \right]\delta \varepsilon \;ds\right)}_{i}\forall \delta \varepsilon \;\mathrm{with}\;\varepsilon =\left(n⊗n\right):\varepsilon$$

where A=a×a is the area of 𝛺□ and $n_{i}$ is the number of 1D elements identified from the image analysis. It is further assumed that there is perfect bonding between the GnPs and the polymer, whereby the normal GnP strain ε is affine with the straining of the polymer, i.e., ε=(n⊗n):ε. Moreover, in Equation (1) the sub-index *m* relates to quantities at the macro-level, whereby $\sigma_{m}$ is the homogenized macro stress and $\delta \varepsilon_{m}$ is the virtual strain at the macro level. On the micro-level, **σ** is the microscopic stress of the polymer, *N* is axial force of the 1-D resolved GnP, cf. Figure 2, and **ε** is the strain tensor at the micro level.

In order to link the macro- and micro-stress responses, the strain at the micro-level ε is subdivided in terms of a subscale strain $\varepsilon_{s}=\varepsilon \left[u_{s}\right]$ defined by $\varepsilon =\varepsilon_{m}+\varepsilon \left[u_{s}\right]$, where $u_{s}\left[x\right]$ is the fluctuating displacement field of the subscale. Upon combining this with Equation (1), the homogenized macro-stress of the GnP enhanced polymer is obtained as

(2) $$\sigma_{m}=\frac{1}{A}\left(\int_{\Omega_{□}}^{}\sigma dS+\frac{1}{l}\;\sum_{i=1}^{n_{i}}{\left(\int_{I}^{}Nn⊗nds\right)}_{i}\right)$$

corresponding to self-balancing of the micro-field

(3) $$\int_{\Omega_{□}}^{}\sigma :\varepsilon \left[\delta u_{s}\right]\;dS+\frac{1}{l}\;\int_{I}^{}N\;\varepsilon \left[\delta u_{s}\right]ds=0\;\forall \delta u_{s}$$

In addition, the condition for kinematic macro-homogeneity can be written as

(4) $$\varepsilon_{m}=\frac{1}{A}\int_{\Omega_{□}}^{}\varepsilon \;dS\Leftrightarrow \frac{1}{A}\int_{\Omega_{□}}^{}\varepsilon_{s}dS=\frac{1}{A}\int_{\Gamma_{□}}^{}u^{s}⊗n\;ds=0$$

where $\Gamma_{□}$ is the boundary of the region $\Omega_{□}$ as shown in Figure 1. Upon assuming Dirichlet boundary conditions $u^{s}=0$ along $\Gamma_{□}$, indeed the kinematic macro-homogeneity is fulfilled. 

The balance relations in Equation (3) is solved using finite elements, approximating the fluctuation field $u^{s}\left[x\right]$ so that the polymer bulk is discretized using standard 2-D bilinear elements. To kinematically couple the 1-D elements to the bulk, the GnP structures are located at the common face between two adjacent bulk elements, cf. Figure 1g. In this development, elastic response is assumed for the bulk and the 1-D elements so that

(5) $$\sigma =E:\left(\varepsilon_{m}+\varepsilon_{s}\right)\;\mathrm{with}\;E=\frac{E}{1+\;\nu }\left(I+\frac{\nu }{1-2\nu }1⊗1\right)$$

(6) $$N=E_{GnP}\left(\varepsilon_{m}+\varepsilon_{s}\right)\;\mathrm{with}\;\varepsilon_{s}=\left(n⊗n\right):\varepsilon_{s}$$

where E and ν are the modulus of elasticity and Poisson’s ratio of the bulk and $E_{GnP}$ is the axial stiffness of the 1-D elements. Please note that $\varepsilon_{m}=\left(n⊗n\right):\varepsilon_{m}$ is the given macroscopic strain and $u^{s}$ is the FE-resolved microscale displacement field from the balance relations in Equation (3). As alluded to in Equation (4), Dirichlet boundary conditions are selected for $u^{s}$.

The elastic modulus of the GnP ($E_{f}$) is used to calculate the axial stiffness

(7) $$E_{GnP}:=l\;t\;E_{f}$$

whereby $E_{GnP}$ is defined in terms of the average depth *l* through the RAE plane, cf. Figure 2, and the average thickness *t* of the GnPs. In order to validate the procedure problem, the stiffness properties E, ν, and $E_{GnP}$ are compared with the experimental measured data. Current results are in the form of basic stiffness response of components E and ν. It should be noted that $E_{GnP}$ has another dimension as compared to E.

For the homogenization, the mesh was auto-created with the extracted GnP from the images mapped onto 2-D surfaces using the Abaqus CAE [25] surface meshing tool (Figure 1g). The mesh for the matrix, GnPs, and boundaries were then assigned to sets using scripts that were developed to be suitable for running in batch format for large data sets. From parameter studies using the computational homogenization, it was noted that GnPs with lengths less than 2 µm had a negligible effect on resultant stiffness during the homogenization procedure and were therefore omitted. Unit displacement was applied in the vertical direction, the horizontal direction, and in shear in order to extract the corresponding homogenized material response. An example can be seen in Figure 1h of the deformation due to the applied extension in the vertical direction. The effective elastic stiffness of the matrix can then be used for component design of e.g., carbon fiber reinforced polymers (CFRP)s, via upscaling the matrix to the ply level response using standard methods.

### 3.3. Computational Homogenization of Thermal Properties

#### 3.3.1. Thermal Conductivity of Constituents

The thermal conductivity of a composite material depends primarily on the thermal properties of the constituents including thermal diffusivity (*a*), density (*r*), and specific heat ($C_{p}$). Partial effect of length, dimension, and structural distribution of reinforcement influence the overall composite materials response. Generally, higher conductivity in LDPE is associated with higher crystallinity of the polymer, where lower conductivity is associated with higher amorphous fraction of the polymer [26]. Graphene in a single layer has been tested to have a thermal conductivity of between 3000 and 5000 W m^−1^ K^−1^ at 23 °C [27]. In this approach thermal conductivity of the constituents were assumed to be constant in the range of temperatures studied. This is a simplification and further studies are needed to verify that this assumption is valid.

#### 3.3.2. Approximation of the Kapitza Effect

Molecular dynamics simulations and FEM analysis have previously been used to study the contribution of Kapitza effect, or interfacial thermal resistance in relation to the effective thermal properties of the nanocomposites, e.g., see [28,29,30]. The scale of observation appears significant, with the Kapitza effect being more pronounced at nanoscale, compared to microscale [31], in agreement with analytical models that do not take into account the interfacial thermal resistance [32]. As a result of phonon scattering, the thermal conductivity of graphene was found to reduce up to 30% [33]. Thus, combining molecular dynamics estimations of the interfacial thermal resistance with FEM analysis provided more accurate estimation of thermal properties [30], at the expense of computational effort.

#### 3.3.3. Computational Homogenization of Thermal Properties

In order to homogenize the effective thermal conductivity of the nanocomposite, a finite element-based heat transfer analysis was performed. Following the developments in [34], it appears that the effective heat flux $q_{m}$ vector for stationary heat transfer homogenize as

(8) $$q_{m}=\frac{1}{A}\int_{\Omega_{□}}^{}qdS$$

where q is the heat flux vector of the micro level. It is determined by the fundamental Fourier’s law, i.e., for isotropic thermal conduction we have

(9) q=−k ∇T

where T is the temperature and k is the coefficient of isotropic thermal conductivity.

Completely in line with the homogenization of the stress response in the previous sub-section, the heat flux is resolved from the microscopic temperature gradient ∇T, where the link to the subscale temperature $T_{s}$ is given by

(10) $$\nabla T=\nabla T_{m}+\nabla T_{s}$$

corresponding to the self balancing of the micro field defined as

(11) $$\int_{\Omega_{□}}q\cdot \left(\nabla \delta T_{s}\right)dS=0\;\forall \;\delta T_{s}$$

**Figure 3:**
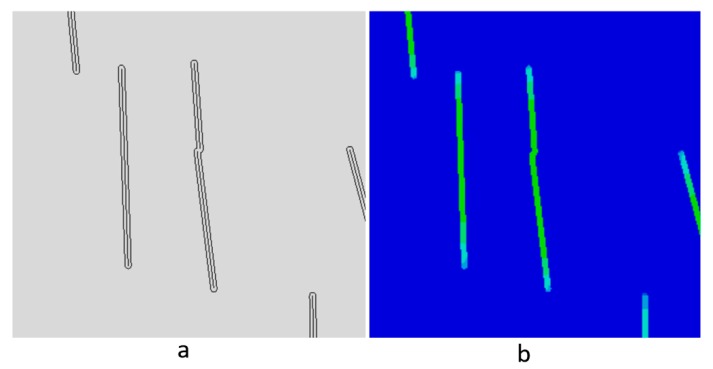
Thermal conductivity homogenization: (**a**) 1-D inclusions with mapped high conductivity region boundaries, (**b**) map of heat flux in the vertical direction.

Note that $\nabla T_{m}$ is the given macroscopic temperature gradient and $T_{s}$ is the FE-resolved subscale temperature field form the balance relations in Equation (11). Like in the stress homogenization, we choose Dirichlet boundary conditions for the subscale temperature $T_{s}$. In order to capture the microstructure with the embedded GnPs, two discrete regions of thermal conductivity are defined. A simple approximation of the microstructure is obtained by creating an interface boundary at the edge of the GnPs and utilizing a step function for the conductivity. The GnP region of high thermal conductivity is assigned an interface conductivity $k_{f}$. The remaining neat resin matrix is assigned the matrix conductivity $k_{m}$. In order to define this region of high thermal conductivity influenced by the GnP, the total thickness of region is approximated at 0.184 µm (Figure 3). The Kapitza resistance for the GnP/LDPE is not known and therefore this resistance boundary was omitted. However, an approximation of the interface thermal resistance can be accounted for by reducing $k_{f}$. This can be done quickly and lends well to the stochastically based large image set processing method presented.

Using the boundary-based approach any overlapping regions of high thermal conductivity are combined into one region as seen in the center of Figure 3. This would simulate the physical phenomenon where the graphene particles of the overlapping regions are in contact. This overlapping is rare in the studied material with only 3% of the images having overlapping regions of high conductivity and therefore having a small impact on the overall results. However, for less aligned nanocomposites it could have a larger impact on the overall thermal conductivity response.

**Figure 4:**
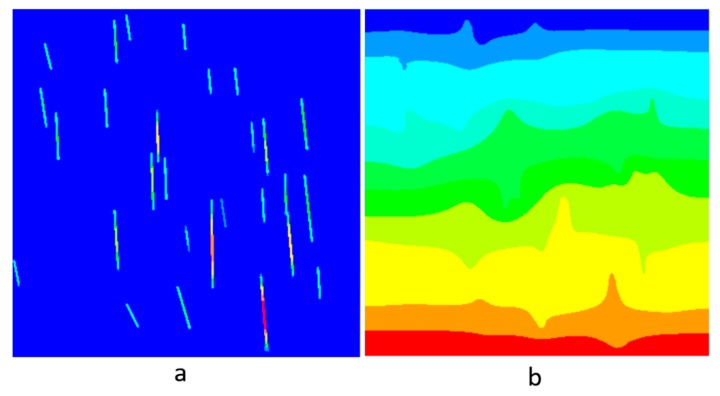
Homogenization of the “vertical” heat conduction parameter k_11_ for the LDPE/GnP nanocomposite: (**a**) heat flux map in the vertical direction, and (**b**) temperature map in the vertical direction.

The heat transfer in the composite was analyzed using the commercially available finite element code Abaqus^TM^ [25] using the two-dimensional diffusive heat transfer DC2D3 and DC2D4 elements. The through-thickness thermal conductivity was computed by prescribing a unit difference in surface temperature between the upper and lower boundaries for the vertical (extrusion) direction and the left and right boundaries for the horizontal (orthogonal) direction. The resulting field was averaged to gain the overall composite thermal conductivity $k_{c}$. A visualization of the heat flux field generated by the computational homogenization which was used to calculate conductivity in the vertical direction can be seen in Figure 4a and the corresponding temperature map can be seen in Figure 4b. An example image sample simulation of 30 × 30 μm is shown in Figure 4. While reducing the number of graphene nanosheets in a graphite nanoplatelet generally increases the overall composite properties, here thinner sheets create more difficulties due to the meshing issues associated with very thin members in a large continuum. For an example image with 1 wt. % GnP, a mechanical homogenization presented in this paper requires 1142 nodes while the thermal conductivity homogenization of the same image requires 4986 nodes. Thinner GnPs would further increase the necessary number of degrees of freedom required to resolve the temperature fluctuation field.

For simplicity only the fiber and matrix phases were included, and the interface was neglected. However, the interface thermal resistance (Kapitza effect) can be accounted for by modifying the highly conductive religions with a reduction factor.

## 4. Results

### 4.1. Computational Results

#### 4.1.1. Mechanical Property Results

The constituent matrix stiffness properties were used for the homogenization procedure. The elastic modulus was found by averaging the E_11_ and E_22_ properties from the experiment resin test performed. An approximate Poisson’s ratio was found from generic LDPE test literature. The constituent properties can be seen in Table 1.

##### Effective In-Plane Stiffness of Graphene

Using the experimental data available for the samples tested in tension along the extrusion direction, the effective multiplier was found via correlation to the experimental observations. The scaled elastic modulus $E_{f}$ of the GnP is

(12) $$E_{f}=f\times E_{m}$$

where E = $E_{m}$ in Equation (12) is the elastic modulus of the matrix and f is a magnification factor. This magnification factor helps account for the invisible GnP known to be present in the nanocomposite among other high aspect ratio platelet phenomena. A number of representative multipliers and their corresponding computational elastic moduli in the extrusion direction can be seen in Table 2. Using the experimental data available for the samples tested in tension along the extrusion direction, the effective multiplier was found via correlation. The effective multiplier obtained to give the best correlation with the experiments for this nanocomposite was f = 1000, corresponding to very stiff GnPs in relation to the polymer bulk.

##### Computational Homogenization of Mechanical Properties

**Figure 5:**
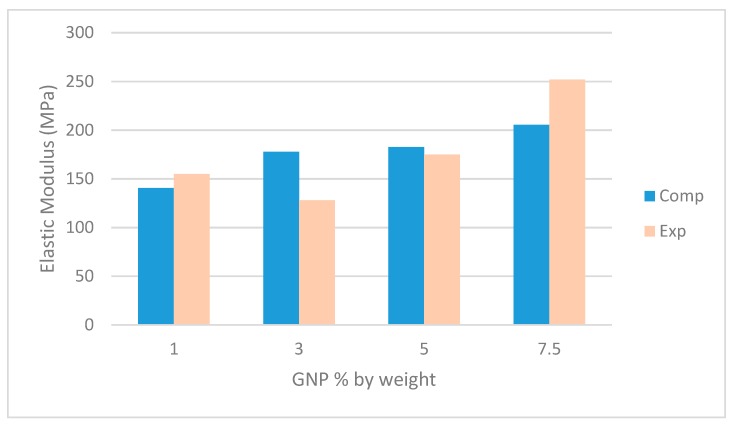
Resulting computational elastic modulus in the extrusion direction, compared with the experimental [1].

The images used in this study for the computational homogenization had graphene concentrations of 1%, 3%, 5%, and 7.5% by weight. The macroscopic stiffness properties were simulated and then compared to the experimental properties of Gaska et al. [18] with respect to increasing volume concentrations, orientation and distribution of the graphene. These images were used to create a 2-D finite element model of the nanocomposite material. In this representation the graphite nanoplatelets are 1-D line features with a higher stiffness than the surrounding matrix. In the results presented here, element piecewise linear representations of the GnPs are obtained since bilinear elements of the bulk were used. The following sample sets were homogenized with the method described in this paper. The results for the samples with a weight fraction of 1% can be seen in Table 3. The resulting homogenized response for the samples with 3% weight fraction can be seen in Table 4. The results for the samples with a weight fraction of 5% can be seen in Table 5. The resulting homogenized response for the samples with 7.5% weight fraction can be seen in Table 6. A summary of the average resulting elastic modulus in the extrusion direction (the direction of nano-structure alignment) can be seen in Figure 5. The resulting average error was approximately 5% when compared to the experimental results for stiffness in the direction of extrusion. Using this method described in the theory section of this paper, the homogenized stiffness was computed for each image sample and then averaged over the full sample set. In general, the error is expected to decrease as the number of samples analyzed increases.

##### Calculation of Volume Fraction of Graphene from Surface Images

**Figure 6:**
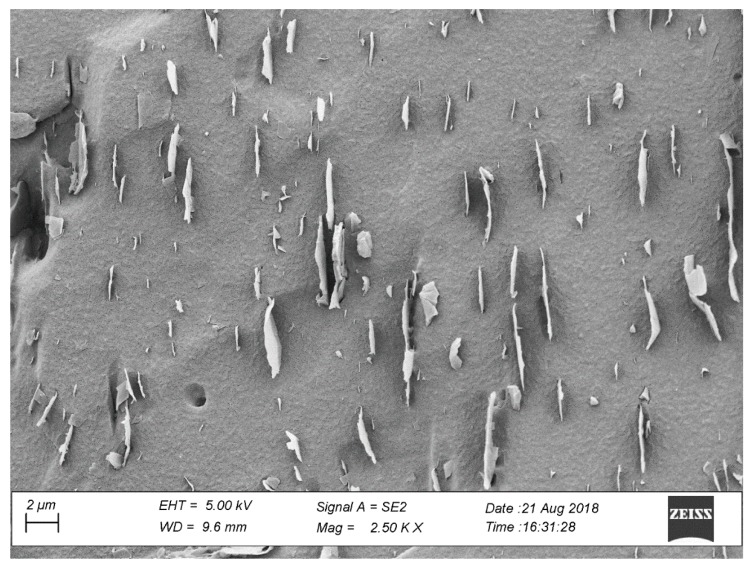
Scanning electron microscope (SEM) image of a 5 wt. % GnP/LDPE nanocomposite.

Figure 6 shows the internal surface of the 5 wt. % GnP/LDPE nanocomposite as seen by SEM. The sections of the graphene nanoplatelets appear as light gray lines with different lengths and thicknesses within the darker epoxy matrix. These SEM micrographs were used as inputs for both finite element analysis and the analytical calculations.

The volume fraction of graphene in a bulk material is dependent on the concentration of graphene in the material. Therefore, calculations will be shown on a representative material with 5 wt. % graphene nanoplatelets, micrographs of the sampled studied. The average number of visible 2D nanostructures found on the surface was 55. The density of graphene ($\rho_{f}$) was estimated to be roughly equivalent to the density of graphite at 2.16 g/cm^3^. The density of the LDPE ($\rho_{m}$) was measured to be 0.926 g/cm^3^. The average GnP length (l) was found from the set of image samples to be 2.376 μm. The average thickness of the GnP (t) was tested to be an average of 7 nm. By using a two-phase diagram with one phase as the matrix and one phase as the GnP, the overall volume fraction of the GnP can be estimated from the weight fraction of the GnP ($W_{f}$) and the constituent densities: the density of the GnP ($\rho_{f}$) and the density of the polymer matrix ($\rho_{m}$).

(13) $$V_{f}=\frac{W_{f}\rho_{m}}{\left(1-W_{f}\right)\rho_{f}+W_{f}\rho_{m}}$$

**Figure 7:**
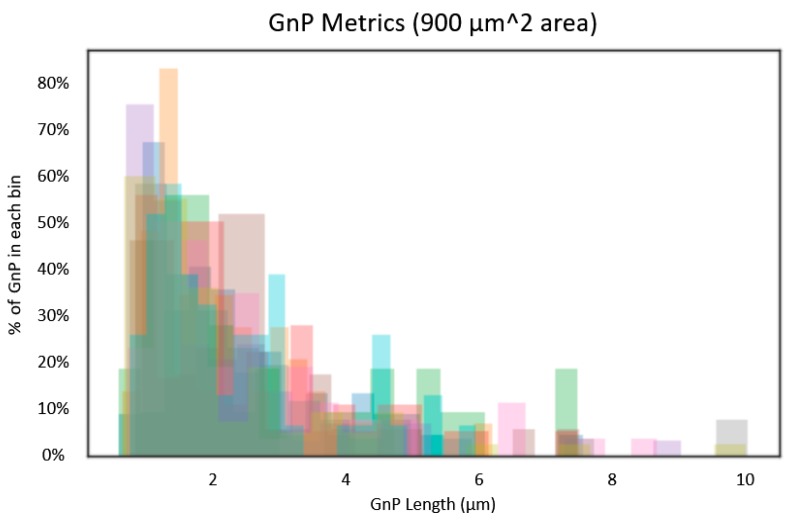
Overlaid GnP Metrics for all 5 wt. % GnP/LDPE nanocomposite image samples.

Using Equation (13), the overall volume fraction ($V_{f}$) of graphene was estimated to be 2.2%. It is important to note that the average length of the GnP is relatively low due to the large number of small GnP in the surface images (Figure 7). From the computational results, it was noted that the longest GnP in the image dominates the effective response. It may, therefore, be advisable to use a GnP length in the analytical method that reflects this understanding of the response.

Figure 7 displays a histogram of the number of GnP in each length bin. This is overlaid for each image processed (i.e., the GnP lengths for each discrete image is displayed as a different color). On the y-axis the percentage of the GnP in each length bin is visible. It can be seen that the peak value is less than 2 μm for every sample which leads to a low average length when compared with the longest GnP.

##### Comparison with Analytical Approaches

It is important to compare the computational homogenization methods with existing analytical approaches in order to compare the accuracy of the results with the experimental results and further understanding of the mechanical and thermal material phenomena which drive the overall composite properties.

Recently, Young et al. [6] developed an analytical approach to calculate the effective in-plane elastic modulus of the graphene nanoplatelets in a given matrix with given GnP characteristics Equation (14). Where $E_{f,eff}$ ranges from $E_{m}$ to $E_{f}$. From there, the micromechanics method based on the rule of mixtures (ROM) is applied to predict the overall effective elastic properties and response of the composite based on this calculated effective in-plane elastic modulus of the graphene or in this case thicker GnP particles. This effective elastic modulus is then used in place of the elastic modulus of the GnP for the subsequent calculations. The effective elastic modulus of the nanocomposite is estimated with the Voigt formulation Equation (15) for the 11 direction (parallel to the GnP) [35]. The Reuss formulation Equation (16) is used to calculate the effective elastic modulus of the nanocomposite for the 22 direction (orthogonal to the GnP) [35]. The Voigt formulation is modified by the Krenchel’s value $\eta_{o}$ = 0.375, for 2D random arrangement for in-plane quasi-isotropic particle arrangement, which is commonly used for ‘flake’ type particle composites [35]. The primary reason this theory is used and not the more common Halpin and Tsai [36,37] based methods is because it is more independent from empirical effects. While these empirically based theories [36,37] do account for the aspect ratio of the particles, the extreme aspect ratio of GnP and further graphene-nanosheets is outside the bounds of the original tests sets and further studies need to be completed in order to assess the effectiveness. This assessment is outside the scope for this paper. The comparison of the homogenized prediction of the overall effective elastic response of the composite can be seen in Table 7. It can be observed that the computational homogenization method can improve upon the initial rule of mixtures based homogenized material response prediction.

(14) Ef,eff≈(lt)2Vf12×(1+νm)×Em

(15) $$E_{c}=E_{f,eff}V_{f}\eta_{o}+E_{m}V_{m}$$

(16) $$\frac{1}{E_{c}}=\frac{V_{f}}{E_{f,eff}}+\frac{V_{m}}{E_{m}}$$

#### 4.1.2. Thermal Property Results

##### Thermal Conductivity of Constituents

The thermal conductivity used for the LDPE resin matrix ($k_{m}$) was approximated to be isotopically 0.37 W m^−1^ K^−1^ at23 °C. This thermal conductivity was found by averaging the extension (K_11_) and transverse (K_22_) thermal conductivity properties from the experiment resin test [18] The thermal conductivity of 1000 W m^−1^ K^−1^ was estimated as the effective thermal conductivity for the present GnP [38]. These material constituent thermal properties can be seen in Table 8.

##### Computational Homogenization of Thermal Properties

**Figure 8:**
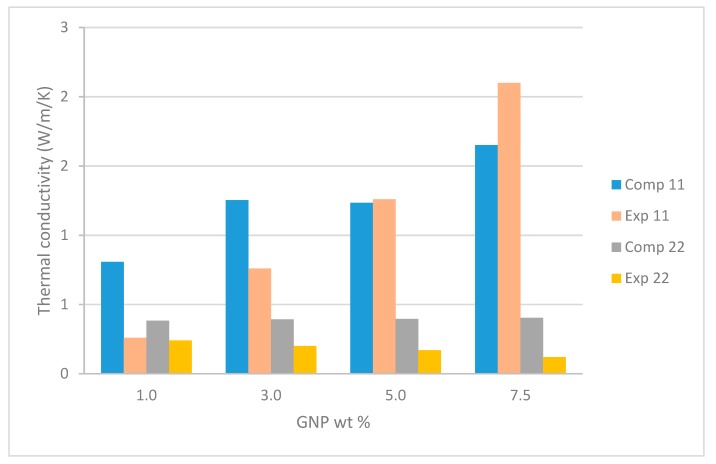
Resulting computational thermal conductivity in the extrusion direction (11) and the transverse direction (22), compared with the experimental [1].

The same representative images were used for computational homogenization of thermal properties as for mechanical properties with graphene concentrations of 1%, 3%, 5%, and 7.5% by weight. Thermal conductivity properties were simulated and compared to the experimental properties of Gaska et al. [1] with respect to the varying GnP concentrations, orientation, and distribution of the graphene. The previously created 2-D representation of the nanocomposite material was adapted for the thermal formulation. However, the 1-D line representations were replaced by 2-D regions of high thermal conductivity influenced by graphite nanoplatelets. The homogenized thermal conductivity output from the computational models correlate with the experimental conductivity values. A summary of the average resulting thermal conductivity in the extrusion direction (the direction of nano-structure alignment) can be seen in Figure 8. Using the method described in the theory Section 3.3.2, the homogenized thermal conductivity was computed for each image sample and then averaged over the full sample set.

In this paper the Kapitza resistance was not calculated, instead it was intrinsically incorporated by adjusting the effective GnP conductivity in the region described in Section 3.3.2. We know from the experimental results that the thermal conductivity of nanocomposite is greatly reduced, demonstrating that there is a significant effect from Kapitza resistance between the two materials. An estimated reduction of 30% was used to simulate the suspected interfacial thermal resistance for the conductivity of the GnP region of high thermal conductivity ($k_{f}$) [34]. Therefore, $k_{f}$ was set to 700 W m^−1^ K^−1^ in the thermal conductivity homogenization simulations.

## 5. Discussion

The method presented in this paper is extremely computationally efficient with the image analysis tool running at 0.03 s/image, the computer-aided design (CAD) conversion tool running at 0.04 s/image, the mechanical analysis running at 0.11 s/image, and the thermal analysis running at 0.12 s/image on a local computer with an Intel^TM^ i7-7600U CPU running at 2.80 GHz. Here the mechanical analysis includes all three simulations (horizontal, vertical, and shear) and the thermal analysis includes both simulations (horizontal and vertical). The use of the surface images and the simplicity of the model allow for quick processing which lends the method to use in analysis of large data sets.

There are limitations to this surface-based approach. These limitations include the correlation between the 2-D and true 3-D properties, variation of the surface normal in the samples, and image variations throughout the material. The current homogenization approach uses a two-dimensional representation which artificially creates uniform plate structures (of the 1-D nano-structures) running out of plane. This assumption will yield an overly stiff homogenization prediction due to the neglection of the intermittent nature of the real GnP. Some methods were investigated to alleviate this issue with mixed success. The inherent variation in surface images used in this method may have a significant impact on the results. In particular, nanocomposites with significant variation in graphene particle size, particle direction, and particle concentration will likely require more images to develop representative stochastic homogenization results. Fractured experimental samples, such as those used in this paper, can also have high and low areas in the z-axis along the fracture boundary. The issues created by these undulations must be considered when using automated image analysis. In addition, the fracture boundaries will tend to propagate through resin rich regions of the nanocomposite material where the material is inherently weaker. This, in turn, will lead to an under prediction of stiffness from this visual surface-based homogenization method.

Even with these limitations, the model was calibrated such that the homogenized stiffness output from the computational models correlated reasonably well with the experimental stiffness values. The overall stiffness properties of the GnP/polymer composite can then be used as a homogenized matrix in further multiscale simulation with additional composite constituents. This can be used to develop micro and macroscale simulations of continuous or chapped fiber reinforced composites with a graphene enhanced matrix.

The elastic modulus multiplier of 1000 obtained for this composite was higher than expected. This yields an effective in-plane elastic modulus of 1.22 × 10^5^ MPa, which is about 12% of the in-plane elastic modulus of a single layer of graphene. One of the reasons for this high effective in-plane elastic modulus could be the omission, intrinsic to the processing method, of GnPs that are too thin to appear in the images. The GnPs must have a sizable thickness or bend somewhat out of plane during or after fracture to be visible in the images and therefore incorporated into the model. For the detailed images used in this study, any GnPs would need to be at least 21 nm thick to be observed if it is directly perpendicular to the field of view. It is currently unknown what percent of GnP are omitted and further investigation is needed to determine this percentage.

Reviewing the computational effective thermal conductivity shown in Figure 8, it can be seen that in the 22 direction (orthogonal to the GnPs) the conductivity is effectively what is input as the LDPE matrix conductivity. The experimental results are somewhat lower than this. A lower matrix conductivity input would be required to come closer to the results. In the 11 direction (in line with the GnP) the results of each sample image vary up to 1.39 W m^−1^ K^−1^ which indicates that there is a considerable amount of scatter in the images. Local particle length and local misalignment can change the conductivity considerably. It is quite possible that more images will be needed to obtain a representative conductivity than a representative stiffness. The larger difference in the constituent’s thermal properties when compared with the difference in the constituent’s mechanical properties corroborates this concept. In general, the reduced GnP region conductivity of 700 W m^−1^ K^−1^ seems to align fairly well with the results. In order to improve overall conductivity properties of the composite surface functionalization or other thermal interface resistance reduction of the GnPs is required. These surface functionalization methods for optimizing photon transport to minimize backscattering [39] can potentially lead to significant increases in thermal conductivity.

Thermal energy in such polymer composites is transferred through a phonon scattering process. The interface between the polymer matrix and a nanoparticle plays a significant role. A combination of poor contact at the interface, weak bonding, and incompatibility between GnP and polymer matrix leads to the increase of thermal resistance at the interface namely Kapitza resistance [40]. The resistance at the interface is identified with the mismatch between phonons’ vibrational spectra in polymer matrix and GnP. Therefore, in order to predict a more accurate thermal conductivity coefficient the current method could be expanded to include a zero-thickness boundary layer with the Kapitza resistance for the polymer and reinforcement. This Kapitza resistance value is dependent on both the matrix and the reinforcement and must be calibrated for each material combination. Therefore, further testing would be needed to improve the current method. The method could also be expanded to include convection and radiation processes, but these are thought to be less dominating phenomena for calculating thermal conductivity.

## 6. Conclusions

In conclusion, the homogenized material response of the graphene polymer nanocomposite was computed via surface images in a systematic and repeatable way that allows for large sets of data to be used to obtain mechanical stiffness and thermal conductivity for the effective material. With the growing focus on multifunctionally of materials, a graphene/polymer composite was chosen due to the desirable range of properties of the graphene constituent including electrical, thermal, mechanical, barrier, high surface area, and others. Two of these properties were selected for an initial study to develop a stochastically-based predictive model in stiffness and thermal conductivity. This model was developed to gain a more efficient understanding of the increased stiffness due to the interaction of the constituents in the nanocomposite. A finite element-based representation was used to meet this need in modeling the multifunctional properties of these composites. This model was combined with an automated method for extracting 2-D nanoparticle dispersion, orientation, and length that can be used for large sets of samples. The stochastic approach utilizes experimental through-thickness scanning electron microscope (SEM) images of nanocomposites to create virtual representations of the experimental microstructure. These SEM images were analyzed systematically through a series of image analysis techniques used to reduce errors in nano-structure extraction, increase repeatability, and significantly reduce the time require to analyze large sets of samples. In the finite element model, the 2-D graphite nanoplatelets were modeled as internal interfaces that store energy for membrane actions. From the homogenization analysis, the macroscopic stiffness properties were simulated and then compared to experimental results. Using a 2-D image and finite element representation of micrographs of low-density polyethylene (LDPE) embedded with (2-D) GnP obtained via melt extrusion, the mechanical and thermal properties were assessed based on computational homogenization. It was noted that the overall composite properties for a given surface sample are highly dependent on the dominant GnP which possess the greatest length and is the most aligned with the direction of loading for both mechanical or thermal homogenization. This understanding of the length and orientation dependency can be used in the future to further improve current analytical based models for multi-particle GnP composites. In addition, the results show that the overall composite thermal conductivity is highly dependent on the thermal resistance at the interface of 2-D nanoparticles, and this has a significant impact on the overall properties even with highly aligned 2-D nano-structures. The method developed here captures size, orientation, and distribution of the graphite nanoplatelets. The models developed will be used to understand the increased stiffness and thermal conductivity of the reinforced matrix of graphene polymer composite materials.

## Figures and Tables

**Table 1:** Resin properties.

E_11_	125	MPa
E_22_	120	MPa
ν_12_	0.38	

**Table 2:** Elastic modulus multiplier calibration.

Multiplier	10	50	100	500	1000
1 wt. % E_11_(MPa)	137.49	139.87	140.21	140.50	140.54
3 wt. % E_11_(MPa)	166.37	175.12	176.47	177.60	177.75
5 wt. % E_11_(MPa)	171.17	180.09	181.45	182.58	182.72
7.5 wt. % E_11_(MPa)	186.15	200.72	203.10	205.13	205.39

**Table 3:** Computational homogenization results for 1 wt. % reinforcement.

1 wt. %	E_11_(MPa)	E_22_(MPa)	G_12_(MPa)
	136.84	126.35	47.68
	127.97	123.19	45.50
	125.36	122.08	44.83
	134.40	125.34	47.05
	129.60	123.96	45.93
	135.62	124.93	47.20
	133.82	123.70	46.65
	134.31	125.40	47.05
Average	132.24	124.37	46.49

**Table 4:** Computational homogenization results for 3 wt. % reinforcement.

3 wt. %	E_11_(MPa)	E_22_(MPa)	G_12_(MPa)
	139.95	126.35	48.24
	151.26	127.54	50.51
	148.68	125.67	49.70
	140.60	123.94	47.92
	156.29	128.53	51.60
	142.65	126.61	48.78
	156.87	125.22	51.10
	150.40	124.83	49.86
	155.50	132.62	52.20
Average	149.13	126.81	49.99

**Table 5:** Computational homogenization results for 5 wt. % reinforcement.

5 wt. %	E_11_(MPa)	E_22_(MPa)	G_12_(MPa)
	157.07	130.69	52.13
	144.97	127.02	49.27
	154.19	128.17	51.15
	151.81	130.58	51.16
	148.38	125.52	49.62
	157.54	125.24	51.23
	155.72	127.05	51.23
Average	152.81	127.75	50.83

**Table 6:** Computational homogenization results for 7.5 wt. % reinforcement.

7.5 wt. %	E_11_(MPa)	E_22_(MPa)	G_12_(MPa)
	153.83	125.86	50.67
	151.83	123.15	49.82
	157.08	130.70	52.13
	177.74	129.69	55.69
	163.55	129.61	53.11
	149.84	126.04	49.98
	164.39	131.12	53.54
Average	159.75	128.02	52.13

**Table 7:** Result comparison 5% GnP by weight.

	E_11_	E_22_
Experimental Result	175	--
Computational Homogenization	183	123
Analytical Method	131	127

**Table 8:** Thermal conductivities of matrix and reinforcement.

(k)LDPE	0.37	W m^−^^1^ K^−^^1^
(k)GnP	1000	W m^−^^1^ K^−^^1^
